# The role of imaging in defining cardiovascular risk to help cancer patient management: a scoping review

**DOI:** 10.1186/s13244-025-01907-9

**Published:** 2025-02-17

**Authors:** Roberto Farì, Giulia Besutti, Pierpaolo Pattacini, Guido Ligabue, Francesco Piroli, Francesca Mantovani, Alessandro Navazio, Mario Larocca, Carmine Pinto, Paolo Giorgi Rossi, Luigi Tarantini

**Affiliations:** 1 Clinical and Experimental Medicine PhD Program, University of Modena and Reggio Emilia, Modena, Italy; 2 Radiology Unit, Department of Diagnostic Imaging and Laboratory Medicine, Azienda USL—IRCCS di Reggio Emilia, Reggio Emilia, Italy; 3 Department of Medical and Surgical Sciences, University of Modena and Reggio Emilia, Modena, Italy; 4 Cardiology Unit, Department of Specialized Medicine, Azienda USL—IRCCS di Reggio Emilia, Reggio Emilia, Italy; 5 Oncology Department, Azienda USL—IRCCS di Reggio Emilia, Reggio Emilia, Italy; 6 Epidemiology Unit, Azienda USL—IRCCS di Reggio Emilia, 42123 Reggio Emilia, Italy

**Keywords:** Cardio-oncology, Coronary artery disease, Multidetector computed tomography, Radiology

## Abstract

**Objective:**

This scoping review explores the potential role of cancer-staging chest CT scans in assessing cardiovascular (CV) risk in cancer patients. It aims to evaluate: (1) the correlation between non-gated chest CT and the conventional Agatston score from cardiac CT; (2) the association between coronary calcium scores from non-gated chest CT and CV risk in non-oncological patients; (3) the link between coronary calcium assessed by non-gated chest CT and CV events or endothelial damage in cancer patients.

**Methods:**

Three different searches were performed on PubMed, according to the three steps described above. Both original articles and systematic reviews were included.

**Results:**

Many studies in the literature have found a strong correlation between coronary calcium scores from non-gated chest CTs and the conventional Agatston scores from gated cardiac CTs. Various methodologies, including Agatston scoring, ordinal scoring, and the “extent” and “length” methods, have been successfully adapted for use with non-gated chest CTs. Studies show that non-gated scans, even those using iodinated contrast, can accurately assess coronary calcification and predict CV risk, with correlations as high as *r* = 0.94 when compared to cardiac CTs. In oncological settings, studies demonstrated a significant link between coronary calcium levels on non-gated chest CTs and higher CV risk, including MACE and overall mortality.

**Conclusions:**

Radiological assessment of coronary calcium on non-gated CT scans shows potential for improving CV risk prediction.

**Critical relevance statement:**

Non-gated chest CT scans can detect endothelial damage in cancer patients, highlighting the need for standardized radiological practices to assess CV risks during routine oncological follow-up, thereby enhancing radiology’s role in comprehensive cancer care.

**Key Points:**

- Cancer therapies improve outcomes but increase cardiovascular risk, requiring balanced management.
- Coronary calcification on non-gated CT correlates with Agatston scores, predicting cardiovascular risk.
- Routinely performed CTs predict cardiovascular risk, optimizing the management of cancer patients.

**Graphical Abstract:**

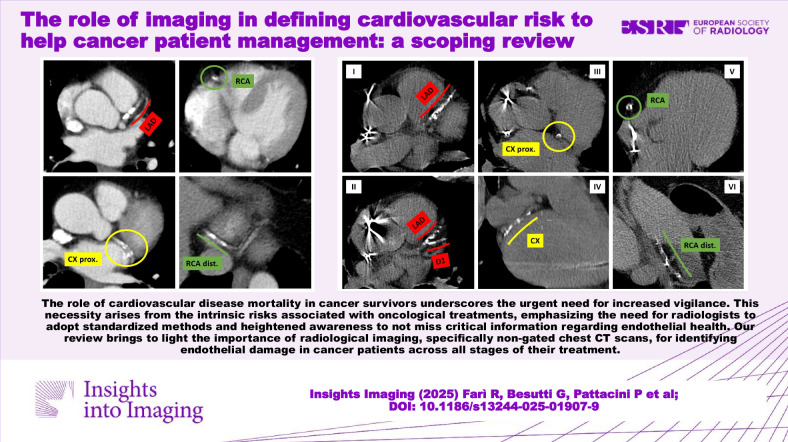

## Introduction

### Advancements in chemotherapy protocols: balancing improved oncological outcomes and cardiovascular competing risk

Advancements in diagnostic techniques and the evolution of oncological therapies over the past few decades have undeniably transformed cancer management, leading to significant improvements in patient outcomes and survival rates [1, 2]. In particular, the emergence of molecular-target and immunotherapy has led to a paradigm shift in management protocols, offering novel and personalized approaches to combat malignancies [3]. However, as underscored by real-world data, the risk of potentially ominous CV complications, such as heart failure, myocardial ischemia, or arrhythmias, has concurrently escalated [4, 5]. The relevance of CV adverse events necessitating a comprehensive, multi-disciplinary approach to patient care [6] is underscored by the temporal analysis of hospital admissions in the USA where one in four patients admitted for CV causes in recent years had mention of cancer [7].

Hypertension, dyslipidaemia, smoking, obesity, and diabetes are frequent in cancer patients. These risk factors frequently co-occur in the same patient and are influenced by both direct and indirect CV effects arising from cancer treatments [8–11]. Cardiovascular toxicity of anticancer therapies encompasses a wide range of conditions [12]. It is now crucial to recognize the risk of inflammatory vascular endothelial damage induced and amplified by oncological treatments, particularly in the coronary arteries [13–16]. Cancer treatments can cause CVD damage either directly, as in the case of anthracyclines [17] or anti-HER2 drugs [18], or indirectly by worsening or amplifying the patient’s intrinsic CVD risk, as with hormonal therapy [19] or immunotherapy [20].

### Complex interplay between endothelial damage and cardio-metabolic alterations in the oncological setting

Recent findings provide compelling evidence for the complex interplay between tumor growth and CV health, corroborating the idea that endothelial damage may be both a cause and consequence of cancer [21, 22]. Recent observations indicate that endothelial dysfunction can predict both future CV adverse events and the future development of solid tumors [23, 24]. One explanation of the underlying mechanism is the role of circulating tumor cells, which can cause direct damage to the endothelium [25]. Also, tumors can secrete cytokines and other pro-inflammatory factors that contribute to systemic inflammation and, ultimately, endothelial dysfunction [26]. Additionally, tumors may promote oxidative stress by generating reactive oxygen species, which can cause direct endothelial injury and subsequent coronary damage [27].

On the other hand, new insights show that an impaired cardio-metabolic state may, itself, be a risk factor for the development of certain tumors [28]. Estimates from the Global Burden Disease Study [29] suggest that nearly 50% of cancer cases are influenced by a combination of environmental, behavioral, and metabolic factors, most of these also being common risk factors for cardiovascular diseases (CVDs), including habits like smoking, alcohol abuse, and the combination of obesity with sedentary lifestyles. The shared risk factors suggest that the association between CVD and cancer is not random but is supported by common pathophysiological processes, probably with chronic inflammation as the common denominator, in line with the “common soil” theory [21, 30].

Recent epidemiological data indicate that a high CVD risk, expressed through a clinical risk scoring system [31] or integrated with laboratory biomarkers [32], not only predicts CVD events but also the development of cancer. A review of the Framingham Heart Study and PREVENT found that cancer patients often had higher smoking, diabetes, antihypertensive medication use, and hyperlipidemia rates [31]. Studies also linked CVD biomarkers like NT-proBNP, HbA1c, and CRP to higher cancer risk and an increased chance of myocardial infarction and stroke [32]. In cardiology, combining clinical scores with biomarkers has proven to improve predictive accuracy, as seen in models like SCORE2 and QRISK3, which better stratify CV risk when biomarkers are included [33, 34]. This approach underscores the potential of integrated models to enhance risk prediction in complex disease profiles. These and other findings in the literature highlight a correlation between poor cardio-metabolic health—commonly seen in conditions such as obesity, diabetes, and hypertension—and an increased risk of various cancers, including those of the pancreas, liver, and endometrium [21, 30, 31]. These associations are primarily attributed to mechanisms such as insulin resistance and hormonal imbalances [35]. For instance, insulin resistance can lead to enhanced tumor growth through chronic hyperinsulinemia. Additionally, obesity can cause excess estrogen production, increasing cancer susceptibility [35]. Dyslipidemia further complicates the picture by promoting chronic inflammation and altering lipid metabolism, essential for cancer cell growth [36]. High levels of oxidized low-density lipoprotein can trigger oxidative stress and activate cancer-promoting pathways, while dysfunctional high-density lipoprotein loses its protective effects against inflammation and oxidation [37].

Given these shared risk factors, it is crucial to acknowledge that cancer patients, especially those with cancers more closely associated with metabolic risk factors, may experience a elevated CVD risk. Assessing this risk in cancer patients is a crucial step in guiding treatment decisions. In this paper we explore available existing evidence on the role of diagnostic and staging imaging in evaluating CV risk.

## Scoping review rationale and aim

The integrated evaluation of radiological and clinical-laboratory parameters has emerged as a critical tool in the attempt to optimize cancer treatment while minimizing the risk of CVD associated with the therapy and the cancer itself.

Recent research suggests that radiological biomarkers could play a significant role in assessing CV risk in patients with cancer [38, 39]. Coronary computed tomography angiography is a well-known method for accurately measuring coronary calcifications using a calcium score, which is a strong predictor of future cardiac ischemic events even when compared to clinical and laboratory parameters [40]. The Agatston score, often used by clinicians to quantify the calcium score, is based on the density and extent of arterial calcifications, calculated by adding the calcium scores of each identified lesion in the coronary arteries [41]. This radiological biomarker has shown predictive value in assessing CV risk by correlating higher calcium scores with increased risk of myocardial infarction and other CV events [42].

Although recent technological advancements have enabled cardiac CT scans to be performed at extremely low radiation doses (averaging well below 1 mSv), there remains concern that even small radiation exposures could increase the risk of cancerous degeneration, particularly in cancer patients [43]. Chest CT scans conducted for other clinical purposes provide a less explored method for obtaining CV risk information for these patients without additional radiation exposure or increased costs. Standard chest CT scans are used for various clinical indications, and the estimation of intra-coronary calcium can also be conducted on these scans, with some studies showing predictive power comparable to dedicated cardiac scans [44–46].

Cancer patients frequently undergo chest CT scans for staging and monitoring purposes, allowing CV evaluation to be integrated into their existing imaging schedule. By leveraging these standard scans, we can explore CV risk in this population without imposing any further radiation dose burden, an approach aligned with patient safety, and optimized use of clinical resources.

This scoping review aims to explore the potential role of radiological imaging, especially non-gated chest CT scans, in identifying endothelial damage in cancer patients. Hence, a three-step literature review has been performed, to assess: (1) the relationship between non-gated chest CT and the conventional Agatston score assessment performed with cardiac CT; (2) the association between CAC score assessments from non-gated chest CT and CV risk in non-oncological patients; (3) the association between coronary calcium assessed by non-gated chest CT scans and CV events or therapy-related endothelial damage in cancer patients.

## Scoping review methods

Three different searches were performed on PubMed, according to the three steps described above. The first strategy aimed to uncover existing studies that elucidate the relationship between non-gated chest CT and the conventional Agatston score assessment performed with cardiac CT for CAC evaluation. Studies evaluating non-gated chest CT performed with contrast administration were included alongside those with unenhanced non-gated chest CT. The second search is aimed at identifying studies that investigate the correlation between coronary calcium assessed from non-gated chest CT and CV risk, mainly gauged by the incidence of CV diseases. Finally, the third search strategy aimed to explore the literature about the significance of coronary calcium assessment using non-gated chest CT scans in cancer patients.

Both original articles and systematic reviews were included, and relevant studies were included in the scoping review. A narrative synthesis of results was adopted. Other details on methods and full search strategies are reported in the scoping review protocol (Supplementary methods).

## Scoping review results

### Relationship between non-gated chest CT and conventional Agatston score

A meta-analysis by Kim et al [45] was identified, reporting the most significant studies on the topic up to 2021. Alongside this meta-analysis, we included a 2014 study by Blair et al (not considered in the meta-analysis) and studies conducted after 2021 (Table 1).

**Table 1:** Summary of studies from PubMed on the relationship between non-gated chest CT and conventional Agatston score assessment

Study	Comparison	Pt. number	Assessment method	Results
Groen et al Am J Cardiol [59]	• Ordinal score on non-gated CT (both non-contrast and contrast-enhanced) • Agatston score on gated non-contrast CT	140	Ordinal scale from 0 to 12, based on the extent in 4 coronary arteries	Strong correlation between ordinally scored CAC on non-gated CT and Agatston score (*r* = 0.94).
Raygor et al JCCT [48]	• Visual score on non-gated CT (both non-contrast and contrast-enhanced) • Agatston score on gated non-contrast CT	934	Visual score for all coronary trees using categories: none, mild, moderate, and severe.	Strong correlation between visual and Agatston scores (Kendalls tau-*b* = 0.76).
Fresno et al Am J Roent [58]	• Ordinal score on non-gated CT (both non-contrast and contrast-enhanced) • Agatston score on gated non-contrast CT	116 *(contrast)* 144 *(non-contrast)*	Ordinal scale from 0 to 12, based on the extent in 4 coronary arteries	Agatston score showed a strong correlation with the ordinally scored CAC on both contrast-enhanced (*r* = 0.94) and non-contrast CT (*r* = 0.93).
Kim et al Kor J Rad [45]	• Ordinal score on non-gated non-contrast CT • Agatston score on gated non-contrast CT	4000 *(from 16 studies)*	Various *(meta-analysis)*	Strong correlation between ordinally scored CAC on non-gated CT and Agatston score (pooled weighted kappa = 0.85).
Blair et al Int J Cardiovasc Imaging [67]	• Ordinal score on non-gated non-contrast 6 mm CT (121 partecipants) • Agatston score on gated non-contrast CT (all partecipants)	228	Ordinal scale from 0 to 12, based on the extent in 4 coronary arteries	The Spearman correlation between the ordinal and Agatston scores was 0.715.
Lee et al Eur Radiol [47]	• Ordinal score on non-gated non-contrast • Agatston score on gated non-contrast-enhanced CT	120	Three methods, with ordinal scale from 0 to 12 and based on 4 coronary arteries: • Extent-based (vessel %) • Length-based (mm) • Density-based (Weston score)	The extent-based method showed moderate agreement with the other two (weighted kappa 0.519), while the Length-based and Density-based methods had excellent agreement (weighted kappa 0.873). The Length-based method correlated best with the Agatston Score (weighted kappa 0.810), whereas the extent-based method had the lowest correlation (weighted kappa 0.570 mm).

Various methodologies have been proposed for evaluating coronary calcium in non-gated chest CT [44, 45, 47–49]. Standard, non-gated, chest CTs, including those utilized for lung cancer screening, have been adapted to perform Agatston scoring, a method traditionally reserved for cardiac-specific imaging [50]. This adaptation is significant, as it bridges the gap between standard imaging techniques and the specialized assessment of coronary calcium. Many studies demonstrate a high level of agreement between non-gated Agatston scores and those obtained from traditional gated scans [49, 51, 52]. Moreover, advancements have led to the introduction of automated techniques for analyzing non-gated scans, and Agatston scores derived from non-gated CT scans have been linked to CV event risks, emphasizing the importance of calcium scoring in predicting patient outcomes [53–56].

Another method suggested in the literature for assessing CAC in non-gated chest CT scans is the use of ordinal scoring which offers a simplified yet effective approach for assessing the burden of coronary calcium [44, 45, 47, 49]. This method employs a basic integer scoring system that correlates with the overall calcium load within the coronary arteries, providing a pragmatic alternative to the more complex Agatston score. Despite its simplicity, ordinal scoring has demonstrated prognostic significance in several studies, correlating well with the risk of CV deaths [57] and showing excellent agreement with traditional Agatston score categories [44, 45, 47, 49].

One widely proposed approach is the “extent method,” which evaluates the four major coronary arteries by the extent of calcification, assigning scores of 0 for no CAC, 1 for less than one-third, 2 for one-third to two-thirds, and 3 for more than two-thirds of the artery’s length showing calcification [44, 47]. The cumulative score, spanning from 0 to 12, is then classified into four severity categories: absent (0), mild (1–3), moderate (4–6), and severe (7–12).

Another reported approach, the “length method”, allocates scores based on calcification length: 0 for no CAC, 1 for < 3 mm, 2 for 3–5 mm, 3 for 6–11 mm, 5 for 12–25 mm, and 9 for > 25 mm, with totals from all major arteries defining severity as absent (0), mild (1–3), moderate (4–8), and severe (≥ 9) [47]. Moreover, the scientific community has delved into a plethora of assessment techniques for coronary artery calcification in non-gated CTs, from straightforward visual evaluations to complex segmented analyses, encompassing detailed investigations into plaque density, automated detection algorithms, and the study of calcification morphology, illustrating the complexity and the expertise required to accurately evaluate coronary artery calcification [44, 49].

Recent analyses also show that using an iodinated contrast medium does not pose a significant barrier to visually estimating coronary calcium, particularly if the scan is conducted during a balanced contrast phase [48, 58, 59]. The balanced contrast phase refers to a particular timing in the delivery of the contrast medium where the contrast material has reached a level of equilibrium within the bloodstream, leading to a relatively homogenous distribution within arterial and venous blood vessels. There are many reasons why iodinated contrast medium doesn’t pose a significant barrier to visually estimating coronary calcium during this phase. First, calcified plaques have a high attenuation value on CT, typically over 130 Hounsfield Units (HU). Iodinated contrast medium usually measures between 100–400 HU, depending on the concentration and phase of the contrast. During the balanced phase, since the concentration of iodine is more homogenous and lower, it doesn’t usually obscure the calcium [60]. Additionally, radiologists can emphasize the differences in attenuation by adjusting the visualization windows on the CT images, improving the display of various tissue densities. By choosing the appropriate window settings, the high-density calcium can be easily visualized separately from the iodinated contrast material [61]. Furthermore, advanced post-processing techniques allow the radiologist to selectively visualize or suppress certain tissue densities. This can help to overcome any interference caused by the contrast medium, allowing for clear visualization of the calcified plaques. Numerous studies have established a tight link between the evaluation of coronary calcium in contrast-enhanced, non-ECG-gated chest CTs and the Agatston score determined through non-contrast, ECG-gated standard cardiac CTs. Groen et al identified a robust correlation between CAC scores using the “extent method” and the Agatston scores from standard cardiac CTs (*r* = 0.94) [59]. Similarly, Fresno et al reported consistent findings, demonstrating a strong correlation between the Agatston score from cardiac CT and CAC scores assessed using the “extent method” in both contrast-enhanced (*r* = 0.94) and non-contrast CT scans (*r* = 0.93) [58].

### Association between coronary calcium assessed from non-gated chest CT and cardiovascular risk or damage in non-oncological patients

Studies collected in the non-oncological setting are reported in Table 2. Regarding the prognostic power, Fresno’s research also revealed that major adverse cardiac events (MACE) were significantly associated with the Agatston score from cardiac CTs (HR 3,2; CI: 1.4–6.9), as well as with ordinal scores from both contrast-enhanced (HR 4,6; CI: 1.2–17.5) and non-contrast CT scans (HR 3,5; CI: 1.5–7.9). Hooks et al analyzed a collection of non-gated chest CTs, including those with and without contrast, employing the Agatston score even in contrast-enhanced scans, to discover that the extent of coronary artery calcification was a predictor of coronary artery disease (HR 1,14; CI: 1.01–1.28) [56].

**Table 2:** Summary of studies from PubMed investigating the correlation between coronary calcium assessed from non-gated chest CT and cardiovascular risk

Study	Assessment method	Outcome	Pt. number	Follow-up	Results
Fresno et al Am J Roent [58]	• Ordinal score on non-gated CT, from 0 to 12 based on the extent in 4 coronary arteries (both non-contrast and contrast-enhanced) • Agatston score on gated non-contrast CT	• MACE	223	6 y	MACE showed significant associations with Agatston score on cardiac CT (HR 3.2; CI: 1.4–7) and with the ordinal score on both contrast-enhanced (HR 4.6; CI: 1.2–17.5) and non-contrast CT (HR 3.5; CI, 1.5–7.9). The risk of MACE was 14 times higher for severe CAC than for no CAC for both contrast-enhanced (HR 13.5; CI: 1.4–130) and non-contrast CT (HR 14.4; CI: 1.6–130).
Blair et al Int J Cardiovasc Imaging [67]	• Agatston score on gated non-contrast CT (all participants) • Ordinal score on non-gated non-contrast CT, from 0 to 12 based on the extent in 4 coronary arteries (121 participants)	• CVD death	57 *(CVD death)* 171 *(controls)*	9 y	Higher Agatston score was associated with a 57% higher odds of CVD death, whereas a higher ordinal score was associated with a 66% higher odds; both were statistically significant.
Shao et al J Cardiovasc Comput Tomogr [51]	• Agatson score on non-gated non-contrast CT. • Visual assessment on non-gated non-contrast CT, categorized as number of coronary involved (no calcification, 1 vessel, ≥ 2 vessels).	• Event-free survival (death or myocardial infarction)	410	7 y	EFS adjusted HR was 2.6 (CI: 1.0–6.4) for patients with 1–500 AU and 5.3 (CI: 1.9–14.2) for patients with > 500 AU compared to patients with 0 CCS. EFS adjusted HR was 2.0 (CI: 0.6–7.2) for patients with 1-vessel CAC and 3.8 (CI: 1.5–9.7) for patients with ≥ 2 vessels CAC compared to patients with no visible CAC.
Shemesh et al Radiology [68]	• Ordinal score on non-gated non-contrast LDCT, from 0 to 12 based on the extent in 4 coronary arteries	• CVD deaths	8782	6 y	CVD deaths increased with increasing ordinal score category (1.2% with score 0, 1.8% with scores 1–3, 5.0% with scores 4–6, and 5.3% with scores 7–12, *p* < 0.0001). A score of 2 or more was a significant predictor of CVD death because the point estimate of the OR was 2.1 (CI: 1.4–3.1; *p* = 0.0002).
Watts et al Coron Artery Dis [69]	• Visual score on non-gated non-contrast LDCT, based on the length and percent of vessel wall involved, considering 3 vessels (from 0 to 36) • Ordinal score on non-gated non-contrast LDCT, from 0 to 12 based on the extent in 4 coronary arteries	• All-cause mortality • CVD mortality	2000 *(1000 deaths, 1000 controls)*	N.A.	ACM showed significant associations with visual (OR 1.4; CI: 1.3–1.6) and ordinal score (OR 1.5; CI: 1.3–1.6) considered as continuous variables. Considering only higher categories, visual (OR 2.3; CI: 1.7–3.0) and ordinal score (OR 2.9; CI: 2.2–4.0) showed an even stronger association with ACM. CVD mortality showed significant associations with visual (OR 1.8; CI: 1.5–2.1) and ordinal score (OR 1.9; CI: 1.6–2.3) considered as continuous variables. Considering only higher categories, visual (OR 5.3; CI: 3.0–9.5) and ordinal score (OR 7.8; CI: 4.0–14.4) showed an even stronger association with CVD mortality.
Hughes-Austin et al JACC Cardiovasc Imaging [52]	• Agatston score on non-contrast CT (both gated cardiac CT and non-gated 6 mm chest CT)	• All-cause mortality	157 *(death cases)* 494 *(controls)*	8 y	ACM showed significant associations with non-contrast non-gated Agatston score (OR 1.5; CI: 1.2–1.9), comparable to the cardiac CT score. Considering only higher categories, non-contrast non-gated Agatston score showed an even stronger associations with ACM (OR 2.6; CI: 1.4–4.9), comparable to the cardiac CT score.

### Association between coronary calcium assessed from non-gated chest CT and cardiovascular risk or damage in oncological patients

As to the oncological setting (Table 3) we identified studies examining the association between coronary calcium levels and an elevated risk of MACE, alongside overall mortality [53, 55–57]. Furthermore, we delved into studies investigating the link between coronary calcium visible on non-gated chest CT scans and the onset of therapy-induced endothelial damage. This includes a look at cancer therapy-related cardiac dysfunction [54] and the changes in coronary calcium levels observed during chemotherapy compared to baseline assessments [62].

**Table 3:** Summary of studies from PubMed exploring the relevance of coronary calcium assessment using non-gated chest CT scans in cancer patients

Study	Assessment Method	Outcome	Pt. number	Cancer	Results
Gal et al JAMA Oncol [53]	Automatic Agatston score on non-gated non-contrast CT for radiotherapy planning	• MACE • All-cause mortality	15,915	Breast cancer	The risk of MACE increased with increasing Agatston scores (for severe calcification HR 3.4; CI: 2.8–4.2). This association was strongest in patients treated with anthracyclines (HR 5.8; CI: 3.0–11.4) and patients who received radiation boost (HR 6.1; CI: 3.8–9.7). All-cause mortality increased with Agatston score (10.9 deaths per 1000 person-years in patients without CAC; 52.8 deaths per 1000 person-years in patients with Agatston score higher than 400).
Shen et al Circ Cardiovasc Imaging [54]	Manual and automatic Agatston score on non-gated non-contrast CT	• MACE • Cancer therapy-related cardiac dysfunction	1468	Diffuse large B-cell lymphoma (anthracycline chemotherapy)	Compared with patients with a score of 0, patients with a score of 1 to 100 had 2.6 higher odds of CTRCD, and patients with a score > 100 had 5.2 higher odds of CTRCD. The cumulative incidence rate of MACEs was significantly higher among patients with a CACS of 1 to 100 (HR 3.7) or > 100 (HR 7.9) compared to those with a CACS of 0.
Phillips et al Int J Cardiol [57]	• Ordinal score on non-gated non-contrast CT, from 0 to 12 based on the extent in 4 coronary arteries. • Framingham risk score.	• MACE • Event-free survival	256	Breast cancer	Ordinally scored CAC, but not FRS was predictive of MACE (*p* = 0.001 for CAC, *p* = 0.154 for FRS). Ordinally scored CAC but not FRS was predictive of EFS (*p* = 0.015 for CAC, *p* = 0. 584 for FRS).
Koutroumpakis et al Front Cardiovasc Med [55]	Semi-automatic Agatston score on non-gated non-contrast CT	• MACE • Overall survival	193	Non-small cell lung cancer (chemoradiotherapy)	Agatston score was independently associated with MACE (HR 1.04; CI:1.01–1.08). Lower Agatston score was independently associated with longer OS (AU < 100 vs. ≥ 100, HR: 0.61, CI: 0.41–0.90).
El-Sabbagh et al Am J Nucl Med Mol Imaging [62]	Agatston score on non-gated non-contrast PET-CT	• Impact of therapy on Agatston score	112 patients *(both baseline and after-therapy PET/TC)*	Lymphoma (chemotherapy)	Agatston score showed a statistically significant increase from the baseline measurement to the completion of chemotherapy (*p* = 0.003), with an average increase of 35% in the score.
Hooks et al Eur J Prev Cardiol [56]	Agatston score on non-gated CT (both non-contrast and contrast-enhanced)	• MACE	98 *(contrast)* 505 *(non-contrast)*	Breast cancer, lymphoma, sarcoma (anthracycline and/or trastuzumab chemotherapy)	Calcification presence or extent did not independently predict MACE (CI 0.97–1.14).

**Fig. 1:**
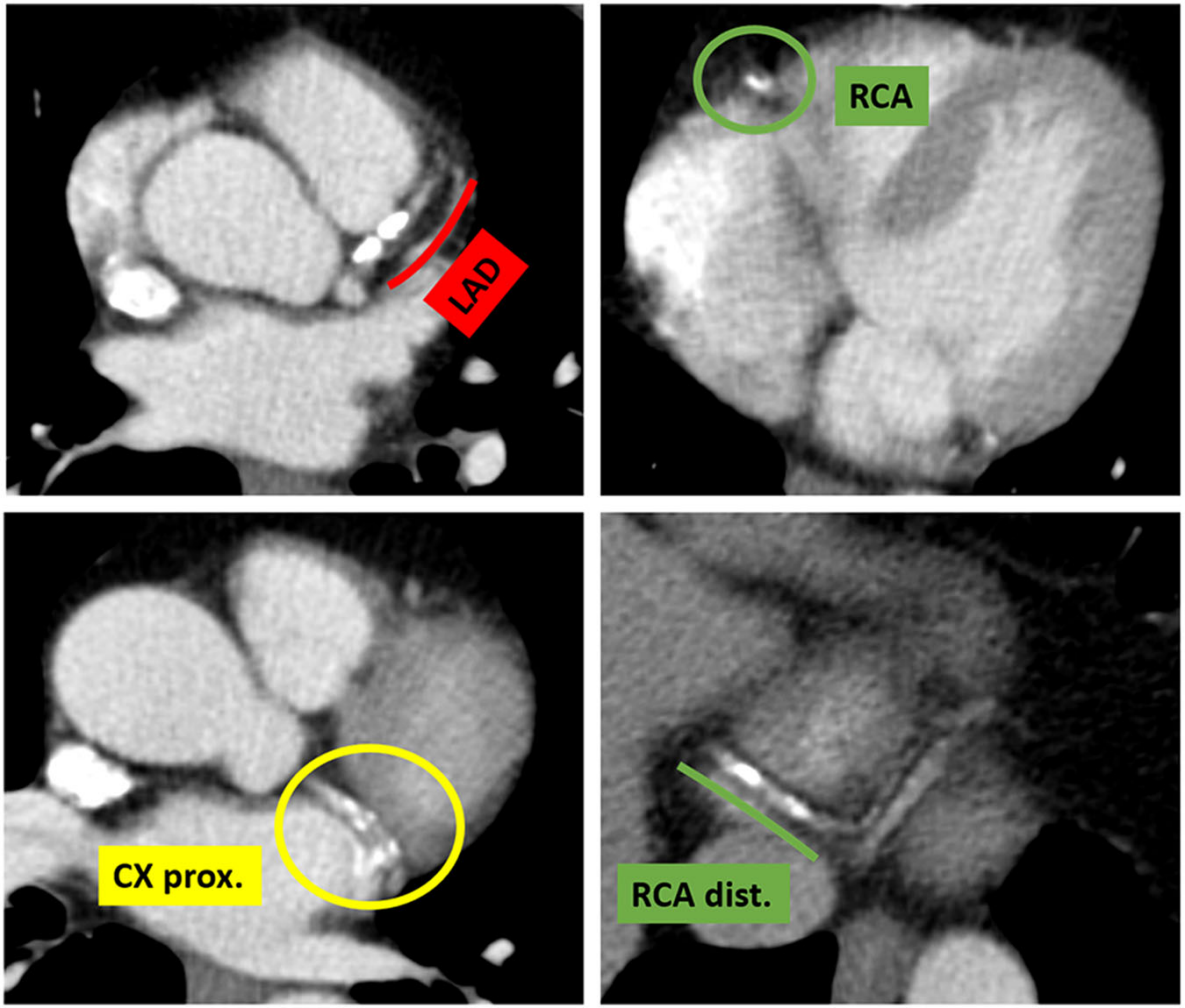
Contrast-enhanced non-ECG-gated CT routinely performed for oncological staging of a 63-year-old man with III-stage non-small cell lung cancer. It is possible to identify extensive calcifications in all the main coronary segments. The patient, who was treated with chemotherapy and radiotherapy, died 7 months later following a myocardial infarction

A significant body of research has confirmed a strong correlation between assessing coronary calcium using non-ECG-gated chest CT scans and elevated CV risk in cancer patients, as we experienced in our center (Fig. 1). For instance, a study by Gal et al involving over 15,000 breast cancer patients found that severe calcifications were associated with a significantly increased risk of MACE, particularly among those treated with anthracyclines or receiving a radiation boost [53]. This study also noted a rise in all-cause mortality with increased Agatston scores. Similarly, research by Shen et al demonstrated that even moderate increases in coronary artery calcium scores were linked to a higher incidence of MACE [54]. Meanwhile, Phillips et al showed that an ordinal scoring method for coronary artery calcium was predictive of MACE and event-free survival even when compared with the Framingham Risk Score [57]. However, a study using contrast-enhanced chest CT found that calcification presence or extent did not independently predict MACE in multivariate analysis [56], highlighting the complexity of these relationships and the need for further investigation.

### Other radiological markers

Besides the reported evidence on coronary calcium, the potential role of imaging in evaluating CV risk and damage in cancer patients may go beyond the coronary region. Given that 6–15% of patients treated with radiotherapy experience radiation-induced valvular changes, other CT-detectable imaging biomarkers could contribute to estimating CV damage in cancer patients [39]. Multiple studies indicate that quantification of aortic valve calcification, a recognized indicator of atherosclerosis, can also serve as a prognostic tool for upcoming CV events, with initial evidence establishing a significant relationship between the degree of calcification and the severity of CV disease [63, 64].

The quantification and characterization of epicardial fat, especially pericoronary fat, thought to be the initial site of inflammation that eventually extends to vessel walls and leads to atherosclerotic plaque formation, might also be valuable [65]. The assessment of pericoronary adipose tissue attenuation using non-gated CT scans has been identified as a potential marker for CV risk. Pericoronary adipose tissue attenuation is associated with coronary inflammation, and its evaluation can provide insights into the local inflammatory status of the coronary arteries [66]. Nevertheless, the practical implementation of systematic estimation of pericoronary adipose tissue attenuation in every CT scan for screening purposes is currently challenged by the substantial time investment required for the analysis. However, as technological advancements progress, the speed of analysis may improve, potentially making it a feasible tool for routine use in the future.

## Implication for practice and research

Assessing CV risk in cancer patients undergoing therapies known to influence CVD risk is essential for optimizing therapeutic management and follow-up strategies. The intersection of CVD and cancer presents significant challenges in clinical practice, requiring a delicate balance between reducing oncologic risk and managing therapy-related side effects. Achieving this balance demands accurate prediction of both oncologic and CV risks, along with an understanding of how these risks are modified by oncologic therapies or mitigated by preventive measures for CVD.

**Fig. 2:**
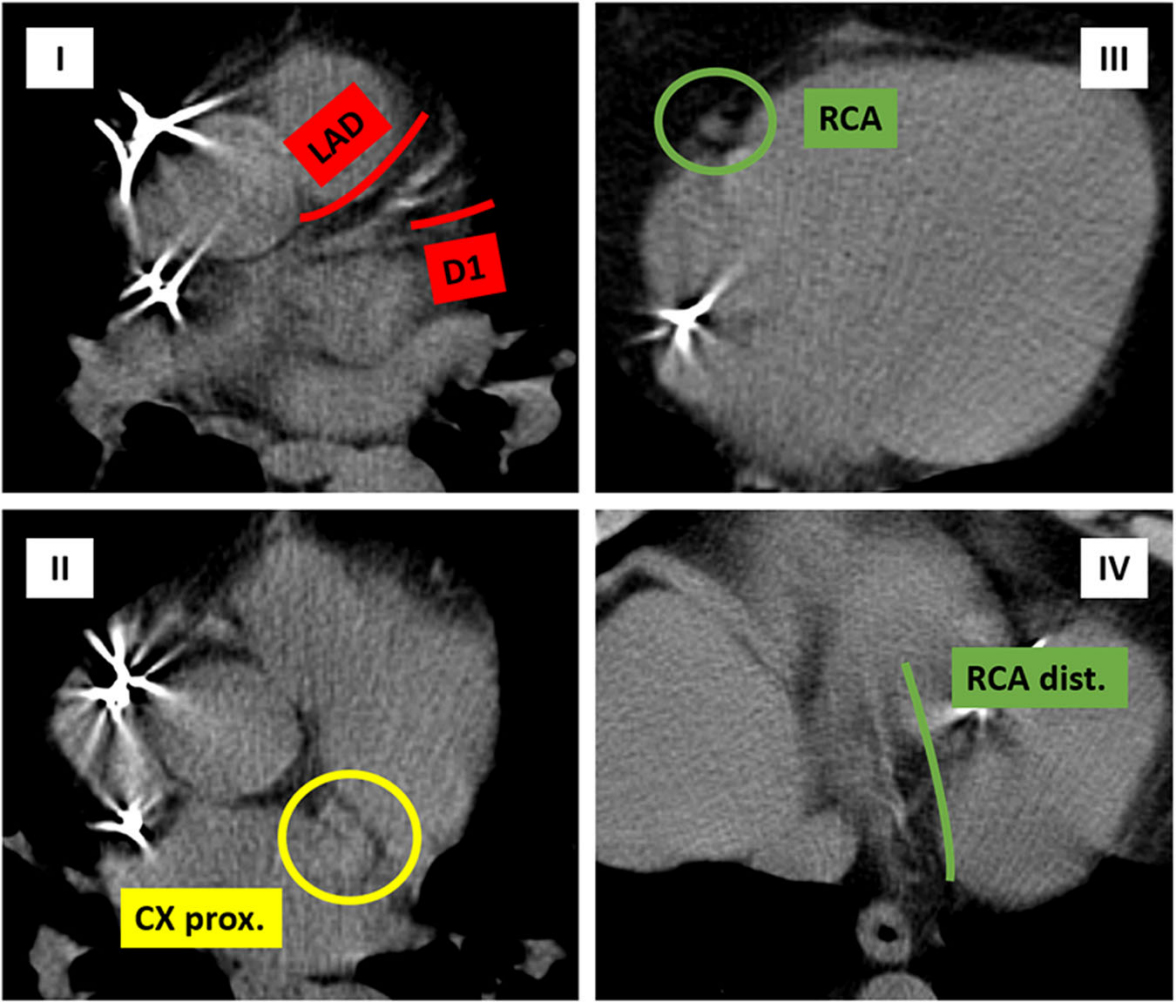
Non-contrast non-ECG-gated CT routinely performed for oncological follow-up of a 74-year-old man with left ventricular systolic dysfunction, kidney failure, and metastatic papillary renal cell carcinoma previously exposed to anthracyclines for former peripheral T-cell lymphoma. The patient came to our attention for cardiological assessment before starting immunotherapy (Nivolumab), due to the failure of Tyrosine kinase inhibitor (Pazopanib) treatment. In 2018, only mild LAD/D1 calcification was detectable (I)

**Fig. 3:**
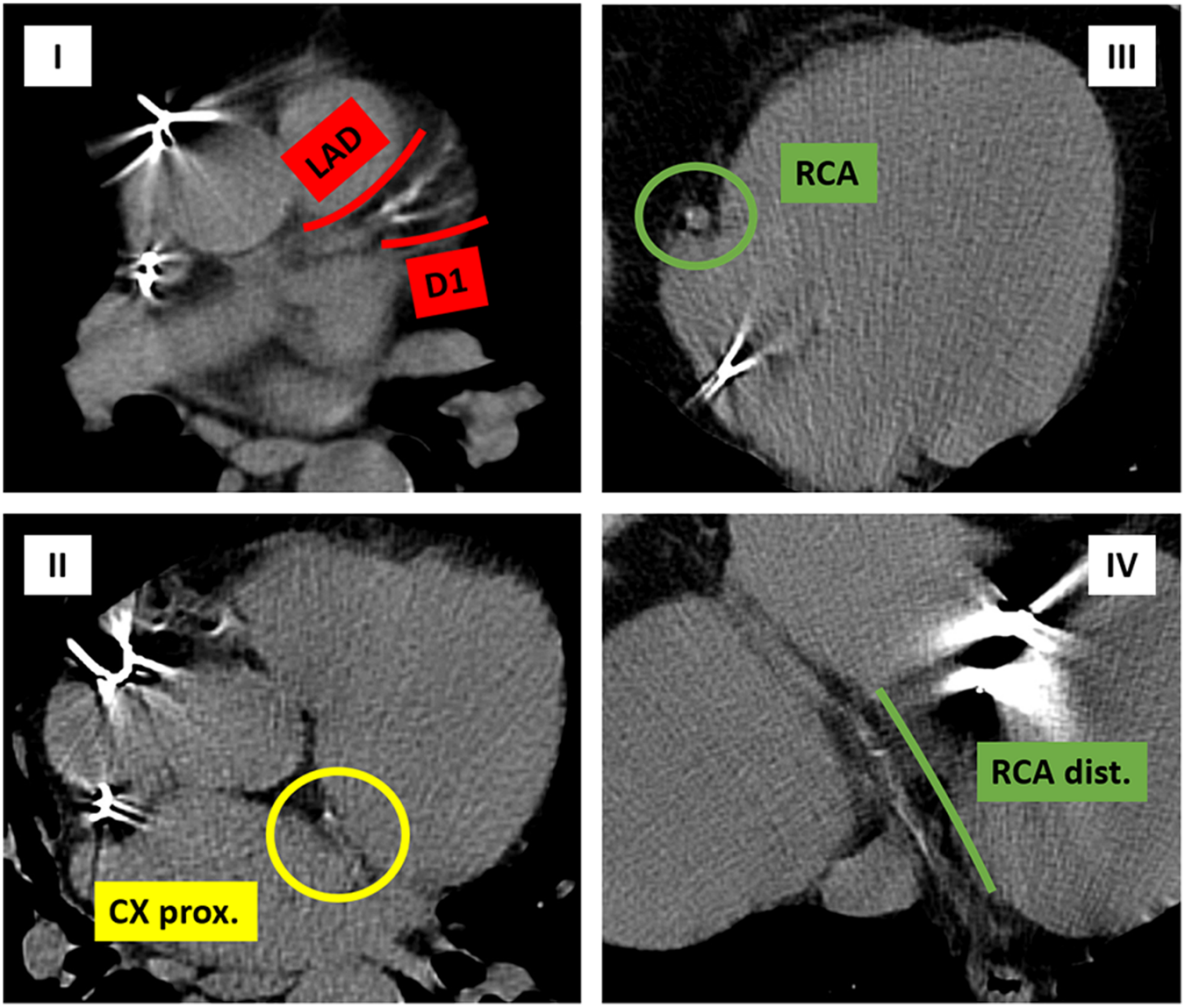
Non-contrast non-ECG-gated CT was routinely performed for oncological follow-up in 2020. Calcific burden increased in LAD/D1 (I) and mild calcifications appeared in CX (II) and DX (III, IV)

**Fig. 4:**
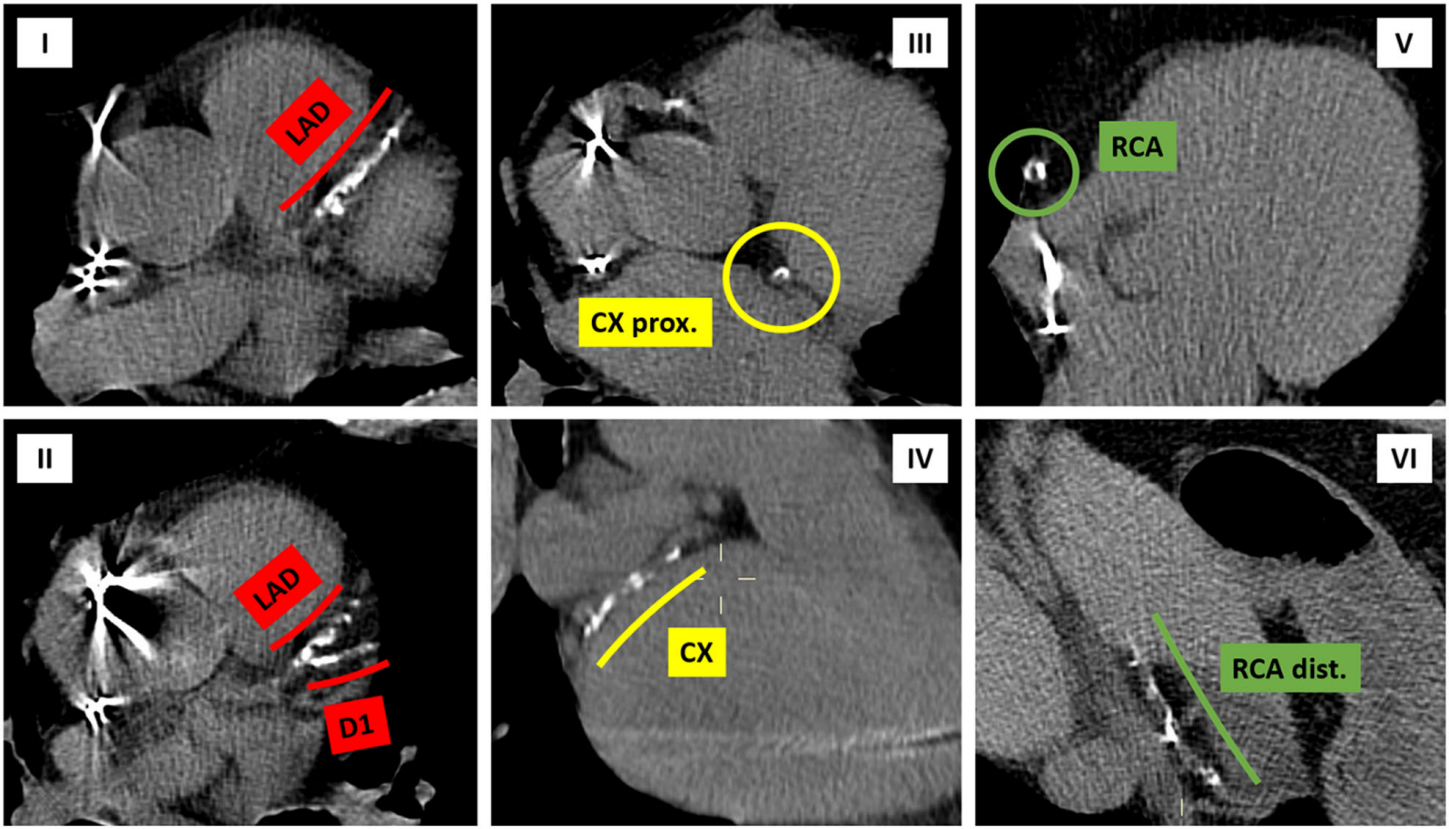
Non-contrast non-ECG-gated CT routinely performed for oncological follow-up in 2022. Calcific burden further increased in LAD/D1 (I, II) as well as in CX (III, IV) and DX (V, VI), leading to severe three-vessel disease. After a multi-disciplinary discussion, an aggressive correction of multiple cardiovascular risk factors (starting Rosuvastatin and Bisoprolol) was performed, and immunotherapy started without any adverse event

Although the results of the studies reported in this scoping review are not univocal, they mostly suggest that radiological assessment of coronary calcium on non-gated CT scan, which cancer patients undergo for oncological reasons, may help CV risk prediction and subsequent patient management, as we experienced in our center (Figs. 2, 3, and 4). To fully realize this potential, targeted education for radiologists and clinicians on assessing coronary calcium and other radiological markers is essential, with a focus on their clinical implications for improving patient care. Strengthening this knowledge base could not only enhance risk assessment but also guide future research to validate the predictive role of these biomarkers in cardio-oncology.

**Fig. 5:**
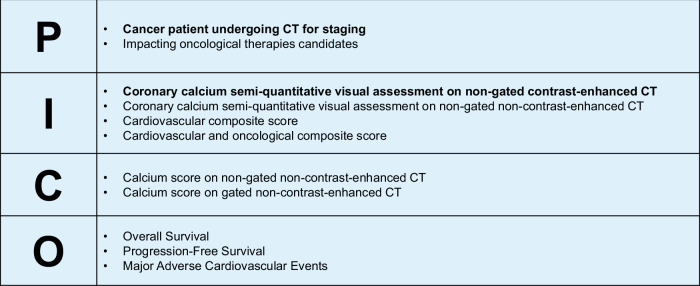
A theoretical PICO (Population, Intervention, Comparison, Outcome) framework addressing the role of semi-quantitative visual assessment of coronary calcium on non-gated contrast-enhanced CT scans in cancer patients

The present scoping review highlights key directions for future necessary research steps. First, studies should assess how semi-quantitative visual assessment of coronary calcium on non-gated contrast-enhanced CT scans correlates with assessments on non-contrast CT and gated CT scans specifically performed for calcium scoring. This comparison is crucial since unenhanced scans are not always performed in cancer patients’ CT for re-staging purposes. Then, comprehensive, large-scale studies are needed to unequivocally demonstrate the predictive power of radiological biomarkers. Moreover, the future of research in cardio-oncology should focus on the integration of coronary calcium and radiological parameters with clinical and laboratory data. Radiological data can be integrated into existing CV risk scores, with the introduction of composite scores including both CV and oncological prognostic factors. Adding clinical parameters, along with other behavioral and metabolic risk factors, could enhance CV risk stratification using information already available at the time of cancer staging and therapy planning. This more accurate assessment of CV risk has the potential to better guide clinical decision-making, enabling more personalized treatment strategies and improving patient outcomes by balancing oncological efficacy with CV safety. To summarize this ambitious research agenda, we included a figure outlining a theoretical PICO (Population, Intervention, Comparison, Outcome) for the clinical question at hand (Fig. 5), which could serve as a guide for designing future original studies and systematic reviews.

Finally, the future goal will be to evaluate if, by coupling endothelial damage assessment with early intervention strategies—such as adjusting oncological treatments or implementing primary/secondary CVD prevention measures—we could eventually change patient relevant outcomes.

Studies included in this review have several limitations that affect their clinical applicability and generalizability. A key challenge lies in the variability of imaging quality and patient cohorts, with differences in tumor types, disease stages, and comorbidities influencing the predictive value of CV risk scores derived from imaging characteristics. One other aspect to consider is the potential for hyperdense contrast media (CM) to mask low-density calcified plaques. These less dense plaques may have attenuation values similar to the contrast agent, leading to challenges in their detection and classification. While CVD is the leading non-cancer-related cause of mortality in oncology patients, other comorbidities also play a role and should be considered. Harmonizing methodologies to ensure comparable and clinically meaningful results while accounting for variability across cancer types and patient populations remains a challenge requiring further collaborative effort. Future research should aim to explore these differences to better tailor predictive models to diverse clinical settings.

In conclusion, our review brings to light the potential of radiological imaging, specifically non-gated chest CT scans, for identifying endothelial damage in cancer patients across all stages of their treatment. The significant role of CVD mortality in cancer survivors underscores the urgent need for increased vigilance. This necessity arises from the intrinsic risks associated with oncological treatments, emphasizing the need for radiologists to adopt standardized methods and heightened awareness to not miss critical information regarding endothelial health.

## Supplementary information

ELECTRONIC SUPPLEMENTARY MATERIAL

## Data Availability

Already published papers.

## Abbreviations

CV: Cardiovascular
CVD: Cardiovascular diseases
HU: Hounsfield Units
MACE: Major adverse cardiac events
