# Correction: The role of imaging in defining cardiovascular risk to help cancer patient management: a scoping review

**DOI:** 10.1186/s13244-025-01981-z

**Published:** 2025-05-15

**Authors:** Roberto Farì, Giulia Besutti, Pierpaolo Pattacini, Guido Ligabue, Francesco Piroli, Francesca Mantovani, Alessandro Navazio, Mario Larocca, Carmine Pinto, Paolo Giorgi Rossi, Luigi Tarantini

**Affiliations:** 1 Clinical and Experimental Medicine PhD Program, University of Modena and Reggio Emilia, Modena, Italy; 2 Radiology Unit, Department of Diagnostic Imaging and Laboratory Medicine, Azienda USL—IRCCS di Reggio Emilia, Reggio Emilia, Italy; 3 Department of Medical and Surgical Sciences, University of Modena and Reggio Emilia, Modena, Italy; 4 Cardiology Unit, Department of Specialized Medicine, Azienda USL—IRCCS di Reggio Emilia, Reggio Emilia, Italy; 5 Oncology Department, Azienda USL—IRCCS di Reggio Emilia, Reggio Emilia, Italy; 6 Epidemiology Unit, Azienda USL—IRCCS di Reggio Emilia, Reggio Emilia, Italy

**Correction to:** **Insights into Imaging**

10.1186/s13244-025-01907-9; published online 17 February 2025

In this article an early version of Fig. 5 was released containing text in Italian. The Italian text has been removed.

Updated Fig. 5 is shown below:

**Figure:**
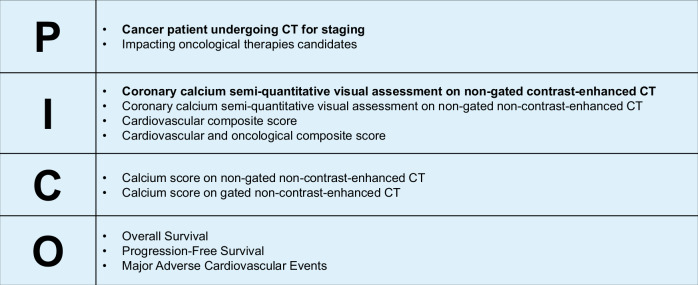

**Fig. 5** A theoretical PICO (population, intervention, comparison, outcome) framework addressing the role of semi-quantitative visual assessment of coronary calcium on non-gated contrast-enhanced CT scans in cancer patients

The original article has been corrected.

