# Supplementary material for: The role of imaging in defining cardiovascular risk to help cancer patient management: a scoping review
Source: Insights Imaging. 2025 Feb 17;16:37. doi: 10.1186/s13244-025-01907-9 (PMC11832977; doi:10.1186/s13244-025-01907-9)
Supplement: Supplementary file 1 — ELECTRONIC SUPPLEMENTARY MATERIAL [file 13244_2025_1907_MOESM1_ESM.pdf]

The Role of Imaging in Defining Cardiovascular Risk to Help Cancer Patient Management: A Scoping Review

ELECTRONIC SUPPLEMENTARY MATERIAL

Question: Can cancer staging chest CT scan help in assessing cardiovascular risk in cancer patients? Steps: 1) Does information from non-gated chest CT correlate with the conventional Agatston score from cardiac CT? 2) Is coronary calcium score from non-gated chest CT associated with cardiovascular risk in non-oncological patients? 3) Is coronary calcium assessed by non-gated chest CT associated with cardiovascular events or endothelial damage in cancer patients?

Objective: To assess the potential role of cancer staging chest CT scan in the assessment of cardiovascular risk in cancer patients.

The three healthcare questions are framed in PICO or PECO as following.

Eligibility Criteria:

- Study design: We will initially include existing systematic reviews of randomised clinical trials or observational studies. To update the existing reviews or to develop a de novo evidence synthesis, in the absence of relevant systematic reviews, we will search for individual studies. Studies reported only as abstracts will not be considered. We will only include studies published in English. Larger and more recent studies will be prioritised.

- Population: Step 1) Individuals undergoing assessment of CVD risk for primary or tertiary prevention; Step 2) Non-oncological patients undergoing non-gated CT for any reason; Step 3) Cancer patients, including all tumor sites, undergoing non-gated CT.

- Intervention (Step 1): Coronary calcium assessment on standard, non-gated, chest CT scan. Different assessment methods will be considered. Specifically, all studies employing quantitative methods, such as the Agatston score, semi-quantitative methods, such as ordinal scales based on the extent of calcifications, and qualitative methods, such as visual scoring systems, will be included.

- Exposure (Step 2 and 3): High calcium score, categorized as reported by the authors.

- Comparison: For Step 1), the comparator is the conventional Agatston score, the gold standard for coronary calcium assessment. For Steps 2) and 3), the considered

comparisons will include the degree of coronary calcium assessed on non-gated CT scans (categorized as low, average, or non-pathological). Additionally, continuous risk increases per unit, interquartile range, or other measures of association will also be included.

-Outcomes: For Step 1), various measures of agreement and association strength between non-gated chest CT-derived calcium assessment and Agaston score on gated non-contrast CT will be considered. For Steps 2) and 3), we will include studies reporting outcomes related to cardiovascular events and survival.

Search strategy: According to the definition of scoping review, the search strategy is aimed at reaching saturation on the topic. Hence, the search will be limited to PubMed[1]. No temporal limits will be used, but larger and more recent studies will be prioritised. Search strategies for the 3 steps are:

1) (“non-gated chest CT" OR "non-gated CT" OR "chest CT" OR “non-gated chest computed tomography" OR "non-gated computed tomography" OR "chest computed tomography") AND ("coronary calcium" OR "calcium scoring" OR "calcium score" OR "Agatston") AND ("cardiac CT" OR "coronary CT" OR "cardiac computed tomography" OR "coronary computed tomography").
2) (“non-gated chest CT" OR "non-gated CT" OR "chest CT" OR “non-gated chest computed tomography" OR "non-gated computed tomography" OR "chest computed tomography") AND ("coronary calcium" OR "calcium scoring") AND ("cardiovascular events" OR "cardiovascular outcomes" OR "cardiac events" OR "mortality" or "MACE").
3) ("non-gated chest CT" OR "non-gated CT" OR "chest CT" OR “non-gated chest computed tomography" OR "non-gated computed tomography" OR "chest computed tomography") AND ("coronary calcium" OR "calcium scoring") AND ("cardiovascular events" OR "outcomes" OR "cardiac events" OR "mortality" or "MACE" or "cardiac dysfunction") AND ("cancer" OR "breast” or “lymphoma” or “chemotherapy” or “radiotherapy” or “sarcoma” or “melanoma”).

Study selection, data extraction and synthesis

Study selection: One reviewer will screen the search results based on the title and abstract and confirm eligibility based on the full text of the relevant articles.

Data collection: One reviewer will extract relevant data from eligible studies on their main characteristics. Extracted data will particularly focus on study design, number of patients

included, patient demographics, methods of calcification assessment, outcomes, and main results.

Synthesis of the results: A narrative synthesis approach will be used to summarize main results, subdivided in the 3 steps. Data pooling will not be considered.

Bibliography

1. EUnetHTA JA3WP6B2-2 Authoring Team. Process of information retrieval for systematic reviews and health technology assessments on clinical effectiveness. Methodological Guidelines. Diemen (The Netherlands): EUnetHTA; 2019. Available from https://www.eunethta.eu/ .
